# Supplementary material for: Patterns of Intron Gain and Loss in Fungi
Source: PLoS Biol. 2004 Nov 30;2(12):e422. doi: 10.1371/journal.pbio.0020422 (PMC532390; doi:10.1371/journal.pbio.0020422)
Supplement: Table S1 — Also available at http://genes.mit.edu/NielsenEtAl/. (4.3 MB ZIP). [file pbio.0020422.st001.zip › NielsenEtAl/html/589.html]

AN7290.1.NCU00348.1.MG05173.1.FG06746.1


```
 CLUSTAL W (1.82) Multiple Sequence Alignments - Introns Inserted


Sequence 1: NCU00348.1	112 aa
Sequence 2: FG06746.1	110 aa
Sequence 3: AN7290.1	108 aa
Sequence 4: MG05173.1	110 aa
Alignment Length: 112 aa
Number Identitical Residues: 94 aa
Alignment Score (without introns) 3894


MG05173.1 	MSRHHP~DLVMCRKQPGIAIGRLCDKCDGKCPVCDSYVRPTTLVRICDECSFGNYQNKCV
NCU00348.1	MSRHHP2DLVMCRKQSGIAIGRLCDKCDGKCPVCDSYVRPTTLVRICDECSFGNYQNKCV
FG06746.1 	MSRHHP2DLVMCRKQAGIAIGRLCDKCDGKCPVCDSYVRPTTLVRICDECSFGNYQNKCV
AN7290.1  	MSRHHP~DLVMCRKQPGISIGRLCDKCDGKCPVCDSYVRPTTLVRICDECSFGNYQNKCI
          	****** ********.**:****************************************:

MG05173.1 	VCGGE0GISDAFYCFECTRLEKDRDGCPKIINLGSSRTD0LFYQKKTNR~TGTY--
NCU00348.1	VCGGE~GISDAFYCFECTRLEKDRDGCPKIINLGSSRTD0RCPGMGLCG~KIVWGG
FG06746.1 	VCGGE~GISDAFYCFECTRLEKDRDGCPKIINLGSSRTD0--IGRQLSI1KNVNHM
AN7290.1  	VCGGE~GISDAFYCFECTRLEKDRDGCPKIINLGSSRTD~LFYQKKSFR~NH----
          	***** *********************************           .
```
